# Therapeutic Options for *Chlamydia trachomatis* Infection: Present and Future

**DOI:** 10.3390/antibiotics11111634

**Published:** 2022-11-16

**Authors:** Rafaela Rodrigues, Lara Marques, Pedro Vieira-Baptista, Carlos Sousa, Nuno Vale

**Affiliations:** 1 OncoPharma Research Group, Center for Health Technology and Services Research (CINTESIS), Rua Doutor Plácido da Costa, 4200-450 Porto, Portugal; 2 CINTESIS@RISE, Faculty of Medicine, University of Porto, Alameda Professor Hernâni Monteiro, 4200-319 Porto, Portugal; 3 Molecular Diagnostics Laboratory, Unilabs Portugal, Centro Empresarial Lionesa Porto, Rua Lionesa, 446 C24, 4465-671 Leça do Balio, Portugal; 4 Hospital Lusíadas Porto, Avenida da Boavista, 171, 4050-115 Porto, Portugal; 5 Lower Genital Tract Unit, Centro Hospitalar de São João, Alameda Professor Hernâni Monteiro, 4200-319 Porto, Portugal; 6 Department of Community Medicine, Health Information and Decision (MEDCIDS), Faculty of Medicine, University of Porto, Rua Doutor Plácido da Costa, 4200-450 Porto, Portugal

**Keywords:** *Chlamydia trachomatis*, genital infection, treatment guidelines, drug resistance, screening, antibiotics, vaccines, infertility, carcinogenesis

## Abstract

Sexually transmitted infections (STIs), such as *Chlamydia trachomatis* (Ct) infection, have serious consequences for sexual and reproductive health worldwide. Ct is one of the most common sexually transmitted bacterial infections in the world, with approximately 129 million new cases per year. *C. trachomatis* is an obligate intracellular Gram-negative bacterium. The infection is usually asymptomatic, notwithstanding, it could also be associated with severe sequels and complications, such as chronic pain, infertility, and gynecologic cancers, and thus there is an urgent need to adequately treat these cases in a timely manner. Consequently, beyond its individual effects, the infection also impacts the economy of the countries where it is prevalent, generating a need to consider the hypothesis of implementing Chlamydia Screening Programs, a decision that, although it is expensive to execute, is a necessary investment that unequivocally will bring financial and social long-term advantages worldwide. To detect Ct infection, there are different methodologies available. Nucleic acid amplification tests, with their high sensitivity and specificity, are currently the first-line tests for the detection of Ct. When replaced by other detection methods, there are more false negative tests, leading to underreported cases and a subsequent underestimation of Ct infection’s prevalence. Ct treatment is based on antibiotic prescription, which is highly associated with drug resistance. Therefore, currently, there have been efforts in line with the development of alternative strategies to effectively treat this infection, using a drug repurposing method, as well as a natural treatment approach. In addition, researchers have also made some progress in the Ct vaccine development over the years, despite the fact that it also necessitates more studies in order to finally establish a vaccination plan. In this review, we have focused on the therapeutic options for treating Ct infection, expert recommendations, and major difficulties, while also exploring the possible avenues through which to face this issue, with novel approaches beyond those proposed by the guidelines of Health Organizations.

## 1. Introduction

Sexually transmitted infections (STIs) have a huge impact on communities; they are associated with individuals’ morbidity and mortality, and also with increased public health expenses through their direct effect on fertility, pregnancy process, and carcinogenesis [1,2]. Chlamydial infection is among the most common curable STIs worldwide, caused by *Chlamydia trachomatis* (Ct) [3]. This bacterium is an obligate intracellular microorganism that preferentially infects epithelial cells, however, it can also infect phagocytes present in the genital tract, such as macrophages [4,5]. It has a life cycle comprised of two distinct forms, the elementary body (EB), which is the infectious form, and the reticulate body (RB), a non-infectious form that is metabolically active [3,6]. The infectious process begins with the contact between the EB and the host cell for the Ct invasion. This contact is established through the major outer membrane protein (MOMP) of the bacterium, triggering molecular pathways that subsequently drive the EB internalization into the host cell [7,8,9]. When in the cytoplasm of the host cell, the bacterium reproductive cycle can start with the EBs conversion into RBs, allowing pathogen replication through binary fission [10]. Finally, RBs differentiate again into the EBs form, to allow their release into the extracellular microenvironment by cell lysis or extrusion [7,11]. Therefore, this immunogenic environment establishment and the biphasic life cycle of Ct seem to facilitate the therapeutic intervention. Nevertheless, Ct has some immune escape evasion mechanisms, interfering with the host’s natural elimination and making infection treatment difficult [12,13,14].

Indeed, the fact that the chlamydial infection is mainly asymptomatic (in more than 60% of men and women), contributes to the frequency of patients being undiagnosed and consequent undertreatment. Consequently, it could be associated with complications and sequels, and the risk of infection transmission rises [15]. Beyond genital infection, it can also cause rectal, oropharyngeal, and ophthalmologic infections [16]. Moreover, an increased risk of co-infection with human papillomavirus (HPV) [17], *Neisseria gonorrhoeae* [18], and *Mycoplasma genitalium* has been reported [19].

According to the World Health Organization (WHO) bulletin on Ct infection prevalence, comparing the 2012 and 2016 available numbers, there was a global decrease in the prevalence of these infections [20]. Notwithstanding, the estimated numbers are still concerning: the WHO anticipated 129 million new Ct infections in 2020 [21]. Therefore, the implementation of Chlamydia Screening Programs, testing asymptomatic women and men, could be one of the key strategies to eradicate this infection [22]. The diagnostic tools available are diverse and are associated with different sensitivities and specificities. In detail, Ct can be detected by culture (not recommended due to the lack of sensibility and the consequent higher incidence of false negative results), enzyme-linked immunosorbent assays (ELISAs), direct immunofluorescence assays, and nucleic acid amplification tests (NAATs). NAATs are the most accurate tests, with specificities and sensitivities higher than 98%, for the genital samples (when non-genital specimens are used, these percentages drop) [23,24,25,26]. Briefly, NAATs must be the gold standard for Ct diagnosis, because the other tests are associated with less sensibility and could result in false negative results, increasing the probability of new infections because of these misdiagnosed cases. Furthermore, NAATs’ methodologies bring an advantage; they can be used with non-invasive test specimens, such as urine and self-collected vaginal swabs, which allied with new, rapid point-of-care diagnostic methods, can diagnose the individuals in around 15 min with a higher rate of accuracy, enabling a “test and treat strategy”; this could be a turning point for controlling Ct infections [27,28,29,30]. In line with this, Herbst de Cortina et al. accomplished a systematic review regarding the performance of these Ct diagnostic tools in order to confront all of these methods and understand which one best fits each country/national health system [31].

Herein, we expose the current state-of-the-art and future treatments in Ct infections, with a particular focus on the standard treatment recommendation guidelines for antibiotics use given by distinct organizations and the potential new therapeutical approaches. Additionally, we discuss the possible mechanisms of antibiotic resistance developed by the bacterium, as well as some strategies to overcome this resistance, using novel drugs development, natural compounds, nanoparticles, and other molecules further detailed in this paper (such as cyclic peptomers, cyclic peptomers, peptide-based inhibitors). Finally, we investigate the promising future directions of this health problem control concerning future vaccination strategies.

## 2. Clinical Presentation of Chlamydial Infection

In the majority of cases, chlamydial infection is asymptomatic [32]. Nevertheless, symptoms could be felt in distinct anatomical regions with different intensities, depending on the bacterium serovar, which is determined based on the specific epitopes of the MOMP encoded by ompA [33,34,35]. Particularly, serovars A, B, Ba, and C are associated with a chronic ophthalmologic disease, designated as trachoma, and blindness; serovars D, Da, E, F, G, Ga, H, I, Ia, J, and K, infect mainly the urogenital tract, resulting in cervicitis in women and urethritis in men and women, or other more complicated outcomes; serovars L1, L2, L2a, and L3, are related with lymphogranuloma venereum (LGV) [6,33,35,36]. The latter are considered the most invasive ones, and if untreated, can lead to rectal fistula or stricture [34]. Indeed, several studies have reported the association between the Ct genotype and the pathogenicity and severity of the infection [36,37,38]. Chen and colleagues have shown that patients with genotype D, the most prevalent in their study, have a low risk of co-infection with other pathogens, as well as a lower association with cervical cancer. On the other hand, genotype F of Ct is mostly associated with bacterial co-infections. Additionally, individuals infected with serovar G share this risk of co-infections. Furthermore, genotype G is associated with mucopurulent cervicitis and cervical dysplasia [39]. Of note, serovar E is commonly associated with co-infection with HPV, a prerequisite for cervical tumorigenesis [40]. Of note, comprehensive studies regarding Ct serovars prevalence revealed distinct geographical distributions, depending on the region studied, the individual’s gender, ethnicity, and sexual orientation [29,36,41,42,43].

Importantly, the authors also indicate that, when not adequately diagnosed and treated, patients may face serious symptoms and consequences, such as pelvic inflammatory disease, ectopic pregnancy, tubal factor infertility, neonatal complications, and other symptoms in different body regions, as shown in Table 1. In fact, persistent Ct infection is also a risk factor for genital tract tumors, demonstrating the urgent need for the screening of asymptomatic sexually active women [36,40,44].

## 3. Current Therapeutic Options

Ct treatment is based on antibiotics prescription. Table 2 presents the drugs recommended by WHO, characterizing their ADME (Absorption, Distribution, Metabolism, and Excretion) profile, mechanism of action, and chemical structure.

Azithromycin is a semisynthetic molecule with antibacterial activity, which is derived from erythromycin [53]. It is a prescribed antibiotic, approved by the Food Drug Administration (FDA) for the treatment of a wide variety of bacterial infections, including Ct. In fact, the WHO recommends azithromycin, as well as doxycycline, as first-line drugs for the treatment of Ct. This molecule can be administered orally, locally (ophthalmological solution), and occasionally, parenterally, depending on the clinical indication. It is highly stable at low pH, increasing its concentration in tissues for long periods of time, compared to erythromycin. The antibiotic activity of azithromycin guarantees that bacterial growth is blocked due to its affinity for the bacterial ribosomes. Specifically, this molecule can infiltrate into the intracellular milieu, binding to the 23S rRNA of the 50S ribosomal subunit of the Ct and inhibiting the assembly of the 50S ribosomal subunit and the translocation step of protein synthesis [54]. Thus, the process of protein synthesis (mRNA, messenger ribonucleic acid, translation) is impeded [55]. Additionally, azithromycin has an immunomodulatory effect that controls the inflammatory process. Drug delivery is mainly at the inflamed tissues, as well as penetrating the phagocytes (leukocytes, monocytes, macrophages, and fibroblasts) allowing it to be effective against Ct [4,5,54].

Doxycycline is part of the class of tetracycline antibiotics, which show biological activity against bacteria through the inhibition of protein synthesis by binding to the 16S rRNA section of the ribosome, inhibiting the binding of tRNA to the 30S bacterial ribosomal subunit. Its administration could be oral or parenteral, depending on the clinical indication. Moreover, there is a reported hepatotoxicity of this drug use [56]. Importantly, following the International Union Against Sexually Transmitted Infections (IUSTI) guidelines, azithromycin is being used as the first-line therapy for this infection in addition to doxycycline. Despite this choice, doxycycline is associated with higher efficacy, and Centers for Disease Control and Prevention (CDC) guidelines recommend a doxycycline regimen as the first treatment, with some exceptions, as detailed further in this section [57,58,59,60]. Reveneau et al. proposed that the different forms of the bacterium, RB and EB, could be responsible for the differences between the drug’s efficacies. In detail, they argue that azithromycin has a better efficacy against the EB form, responsible for persistent infections, whereas doxycycline is more appropriate against the RB form, present in acute infections [60,61].

Erythromycin is an antibiotic of the macrolide class. Its anti-bacterial activity is similar to that of azithromycin [62]. Despite both drugs having the same efficacy against Ct infection, the advantage of erythromycin is its low cost. Nevertheless, it is less safe to use on pregnant women [63].

Tetracycline is a broad-spectrum antibiotic that acts by inhibiting the cell translation process by binding to the 30S bacterium ribosomal subunit. In addition, it can interfere with the cytoplasmic membrane of Ct, affecting the leakage of intracellular content into the extracellular medium. Clinicians must consider that this molecule can cause adverse effects in asthmatic patients [64].

Levofloxacin is a fluoroquinolone antibiotic that can be administered orally. In terms of biological action, its bactericidal activity is through interference with the DNA replication by binding to the key enzyme’s DNA gyrase and topoisomerase IV. Rarely, it has been associated with liver injury [65].

Amoxicillin is an antibiotic whose mode of action is through the inhibition of cell wall biosynthesis, leading to bacterial lysis. This pharmacological compost formulation can be used orally or parenterally. In cases of overdose, individuals can develop hematuria, oliguria, abdominal pain, acute renal failure, vomiting, diarrhea, rash, hyperactivity, and drowsiness [66,67]. In pregnant women, amoxicillin was shown to be a better option than azithromycin in terms of side effects [68]. Moreover, it was verified that it has high efficacy in Ct infection treatment [69].

Tetracycline and povidone iodine are part of the first-line Ct treatment, according to the WHO guidelines. Tetracycline hydrochloride is a semi-synthetical naphthacene antibiotic that inhibits protein synthesis through different mechanisms, including: blocking of the A site of bacteria ribosomes, interruption of the elongation process, inhibition of oligosaccharide side chains attached to glycoproteins, and misreading of the genetic code [70]. In turn, povidone iodine (water-based solution) is an anti-septic agent that can be used locally for ocular prophylaxis immediately after birth [34,71]. Alternatively, silver nitrate is used, which destroys harmful microorganisms or inhibits their activity [72], or chloramphenicol eye solutions, which is a broad-spectrum antibiotic can also be used [73]. Furthermore, it is important to know that depending on the type of Ct infection and the patient’s condition, clinicians must choose an adequate therapeutic strategy in each case. Table 3 synthesized the current therapy strategies recommended based on WHO, IUSTI, and CDC guidelines to effectively treat each type of Ct infection [20,57,58].

Generally, in cases of uncomplicated genital infection, the guidelines highlight that doxycycline is the treatment with higher efficacy and it must be used as first-line therapy, the others are alternative options to use in case of drug contra-indication, resistance, or other reasons [57]. For patients with anorectal infection, they recommend doxycycline orally twice daily for 7 days. The WHO guidelines suggest pregnant women should use azithromycin over erythromycin or amoxicillin. Of note, doxycycline and levofloxacin are contraindicated in pregnancy. For LGV, the recommended treatment is doxycycline; in case of contraindication, azithromycin may be considered. For ophthalmia neonatorum, particularly in conjunctivitis, the use of azithromycin over erythromycin is recommended. Importantly, guidelines recommend topical ocular prophylaxis as an infection prevention measure for all neonates. There are several options for topical application to both eyes immediately after birth [20].

It must be highlighted that treatment options, in some countries, are based on associated costs, rather than on biological behavior, therefore, adverse events may occur [20]. In order to avoid the adverse outcomes of Ct treatment, the therapeutic agents’ properties must be explored, as well as the host infection establishment.

Studies regarding the pharmacological interventions for Ct infection, comparing efficacy and safety of the drugs, are still few and were mainly developed with pregnant women patients, thus potentially biased and not generalizable.

## 4. Treatment Failure and Novel Approaches

The main reasons for treatment failure are poor compliance with treatment, the test of the cure performed too early, and the fact that the partner(s) of the infected ones are not informed and subsequently, not treated, thus they could infect others and re-infect the partner(s) [74]. Additionally, this lack of therapy efficacy can occur due to antibiotic resistance, triggered by gene mutations in the bacteria, or persistence, which occurs in the case that the bacteria are not efficiently eliminated due to their natural features becoming tolerant to the drug [75]. Concerning Ct antibiotic resistance, an in vitro study, including a country with the greatest consumption of azithromycin in Europe, Croatia, did not find azithromycin and doxycycline resistance in the 24 studied samples of urogenital isolates of Ct infection [76]. Nevertheless, an experimental study in the UK, comparing azithromycin with doxycycline, demonstrated a higher treatment failure rate of azithromycin in non-genital infections [77]. Multidrug-resistant Ct serovars may be one of the reasons for azithromycin treatment inefficiency. In detail, some in vitro studies report that point mutations in the ribosomal protein of the bacterium genotype L are responsible for azithromycin resistance [60]. In addition, there is in vitro evidence demonstrating that prior exposure to penicillin could lead to Ct azithromycin resistance [78]. Mestrovic et al. have reported that azithromycin resistance, in vitro*,* could be raised through mutations in Ct 23S rRNA genes [79]. In addition, tetracycline resistance is developed by tet(M) gene mutations [80]. Benamri and colleagues described the fact that fluoroquinolones resistance could be developed via gyrA, parC, and ygeD gene mutations [81].

Antibiotherapy persistence of Ct occurs due to the life cycle of this bacterium [82]. As previously detailed, the Ct life cycle has two distinct forms, with RB being the one that can go through growth arrest in stress cell conditions [6,11,83]. In line with this, several factors can contribute to this pathogen phase persistence, as reported by Mpiga and Ravaoarinoro [84]. Specifically, there is evidence that cytokine, tumor necrosis factor (TNF–α), and interferon gamma (IFN–γ) have an influence in the persistent stage [85,86]; as well as the bacterium growth in non-permissive cells [87]; nutrient limitation [88]; additionally, some antibiotics, such as penicillin, ofloxacin, and ciprofloxacin can interfere with the Ct differentiation stage [78,89,90]. Of note, it must be highlighted that this persistence state phase or heterotolerance is difficult to surpass due to the difficulty in measuring it [75].

Therefore, based on the evidence, there is a need for the development of novel drugs in order to successfully combat Ct infection [10]. Some authors have investigated the role of Corallopyronin A, an antimicrobial compound synthesized by *Corallococcus coralloides*. It acts specifically by binding to a domain of the bacterial DNA-dependent RNA polymerase, inhibiting the growth of Ct [91]. Furthermore, Shima et al. have demonstrated promising outcomes, suggesting it as a future alternative for Ct therapy [91,92]. Additionally, a nanoparticle designated PDGFR-β siRNA-PEI-PLGA-PEG NP, developed by Yang et al., successfully reduces vaginal Ct infection through autophagy induction in human cells, concomitantly with the knock-down of a gene coding of an important surface binding protein of Ct, platelet-derived growth factor receptor beta (PDGFR-β) [93]. Recently, Núñez-Otero and his team, have developed and uncovered the second-generation 2-pyridone amide (KSK213) role in Ct infection control, with reduced toxicity for humans without disturbing the commensal flora. This molecule has revealed its effects in the transcription inhibition of crucial genes responsible for the differentiation from EB to RB, which could be a key control phase of Ct infection [94]. Additionally, there have been efforts to develop natural anti-chlamydial treatments based on extracts. Hamarsheh et al. have investigated in vitro the effect of *Artemisia inculta Delile* extract, which was shown to effectively inhibit Ct infection in HeLa cells [95].

Since 2020, as the antibiotic resistance issue has remained critical, some authors developed studies with potential non-antibiotic weapons. Lam and colleagues have published findings regarding cyclic peptomers as inhibitors of Gram-negative bacteria, and they suggest using 4EpDN cyclic peptomer as a prophylactic treatment against Chlamydia trachomatis due to the strong inhibitor effect that they found in the type III secretion system (T3SS), a virulence factor of the bacteria [96]. Additionally, Hwang et al., optimized peptide-based inhibitors (2-Pyridone-based analogs) in order to better target HtrA serine protease in Ct, an enzyme essential to several bacterial vital functions, which seems to be a promising strategy [97]. Finally, Kazakova and colleagues have defended the need for further research to investigate the promising role ofC-ring oxygen and nitrogen erythrodiol derivatives against Ct infections [98].

Interestingly, drug repurposing, a strategy commonly investigated for cancer treatment, has also been explored in this field [99,100]. Specifically, Itoh et al. have reported the potential role of bortezomib, an anticancer drug, to treat Ct infections by apoptosis induction [100]. Notwithstanding all these new strategies for treating Ct infection, further comprehensive studies are needed in order to improve the translation of these research results into clinical practice.

Indeed, the more effective way to control and eradicate Ct infection is through a vaccination plan that must comprise the individuals before they became sexually active, to maximize immunity, reducing Ct prevalence, and consequently, eradicating the infection. However, Ct vaccine development has proven to be a challenge throughout the years [101]. Brunham and Rappuoli have made assertive conclusions about the barriers to vaccine development, defending the position that there are currently no scientific impediments to this purpose, highlighting recent advances in modern medicine as positive for progress, but also showing that other non-scientific barriers to progress, which do not prioritize this research, have been a negative influence on vaccine advancement. They also propose that the secret to vaccine success is the involvement of four different sectors: the public sector, the clinical sector, industry, and discovery, all working in the same direction [102]. In detail, researchers have been developing different types of vaccines, among these, (1) first-generation Ct vaccines, with an associated biological risk due to their bacterial inoculation origin, (2) the second-generation vaccines, which were designed only using subunits of the bacterium, and the (3) third-generation, more modern than the others, using the pathogen’s DNA [101,103]. Firstly, a first-generation vaccine was evaluated in 1960 to treat trachoma; however, even when adjuvants were used to increase the immune response, immunogenicity was not induced at a sufficient level; re-infections still occurred. Additionally, it was associated with increased inflammation, resulting in the worsening of inflammatory diseases [7,104,105,106,107]. Later, second-generation vaccines were assembled, using subunits of the pathogen as their expressed surface antigens (MOMP) in order to be more effective and safer. Interestingly, this generation of vaccines is already capable of promoting cellular and humoral immunity [108]. Researchers developed oral vaccines using this strategy and tested them in non-human primates and mice. Human clinical trials followed, demonstrating immunoglobulin G (IgG) and immunoglobulin A (IgA) stimulation by the vaccine, as well as other molecules associated with immunity stimulation (IFN-γ). Importantly, these studies have proved the capacity of these vaccines to stimulate antigen-specific immune responses in humans [7,109,110,111]. However, researchers report that it provides limited protection against Ct infection [7,111]. Therefore, the third generation of vaccines was developed using DNA techniques and, in some cases, plasmid vectors carrying the foreign gene of interest. These are more cost-effective and more adequate at triggering humoral and cell-mediated immune responses [7,101]. However, there are several disadvantages associated with this type of vaccine, including the possibility of genome integration and the risk of anti-DNA antibody development [112]. In addition, as Vasilevsky et al. described, even more vaccine approaches have been developed, yet, despite massive efforts, vaccine effectiveness is still not at the levels needed, thus some researchers are focusing their attention on computational strategies [112]. Currently, the efforts are in line with the advances in genomics and bioinformatic tools, in a multi-omics landscape, allowing for an in silico vaccine design that now requires in vitro validation [113,114,115]. Recently, some authors developed a method to create new candidate vaccines, using the biosoftware AllerTOP (Bioinformatics tool for allergenicity prediction. Available online: https://www.ddg-pharmfac.net/AllerTOP/ (accessed on 15 November 2022)) [116]. They studied the predicted epitopes of lymphocytes T and B that could stimulate long-lasting immunity against Ct and concluded that a chimeric peptide will be more efficient. The novel therapeutic epitope vaccine candidates, known as “LSWEMELAY”, “LSNTEGYRY”, “TSDLGQMEY”, “FIDLLQAIY” and “FSNNFSDIY”, described by Shiragannavar and colleagues, must be validated experimentally in order to complement the *in silico* studies to conclude whether the vaccine is efficacious and provides long-term immunity stimulation for translational application [113,115]. In addition, as defended by the authors, a vaccine combining multi-epitopes must be studied because it could be more promising due to the distinct interactions that it could have with the human leukocyte antigen (HLA) molecules [115]. This thesis is also defended by Aslam et al., who developed a study concerning in silico multi-epitope-based vaccine (MEBV) development, concomitantly with an adjuvant (Cholera toxin subunit B) coupled to increase the immune system response because the MEBV itself cannot trigger enough immunogenicity. The authors tested the physio-chemical properties, antigenicity, immunogenicity, allergenicity, secondary structure, solubility, and other important features of this vaccine, using bioinformatic tools, a cost-effective method for the vaccine design, and have concluded that this prototype can successfully stimulate the humor and cell immune responses against Ct. Thus, a forward step is required to test the tolerance, safety, and effectiveness of this MEBV in vitro in future experimental trials in order to approve an effective vaccine [113].

## 5. Conclusions

Ct infection is one of the most common sexually transmitted infections worldwide that could be associated with serious health problems in the genital tract as well as perinatal morbidity of fetuses, even when it runs an asymptomatic course. Therefore, a need for screening measures arises in order to adequately treat the infection according to the guidelines. Ct infection treatments are based on antibiotics prescriptions. Nevertheless, there is a risk of drug resistance and re-infection. Therefore, it is urgent to achieve progress in the development of therapeutic weapons against Ct infection. Indeed, in the future, the key to Ct control must focus on public health intervention through populational screening of asymptomatic individuals to avoid infection transmission and adequately treat patients in a timely matter. Concomitant with this strategy to eradicate the infection worldwide should be the administration of an effective vaccine.

In conclusion, this review highlights the need for a public health intervention with Ct screening to better treat this infection which could have serious complications for human health. Moreover, we reinforce the necessity for further laboratory studies regarding vaccine development and the MEBV approach in order to prove the effectiveness of *in silico* studies and consequently, allow for immunization of future populations, which will only be possible by combining efforts to study potential vaccine candidates, study safety and efficacy within the population, and accelerating cost-effective vaccine manufacture and implementation in order to eradicate this health problem.

## Figures and Tables

**Table 1:** The most common symptoms of Ct infection according to gender, condition, and anatomical region infected [34].

Genital Tract	Symptoms
*Uncomplicated infection*	
Female	Abnormal vaginal discharge; dysuria; post-coital and intermenstrual bleeding
Male	Urethral discharge; dysuria; testicular pain
*Persistent infection*	
Female	Pelvic inflammatory disease; ectopic pregnancy; salpingitis; tubal factor infertility
Male	Epididymitis
Non-genital Tract	
*Rectal infection*	Rectal discharge; rectal pain; blood in the stools
*Oropharyngeal infection*	Pharyngitis and mild sore throat
Pregnancy complications	Preterm birth and low birth weight
Perinatal transmission	Neonatal conjunctivitis and/or nasopharyngeal infection; ocular discharge and swollen eyelids

**Table 2:** ADME profile, mechanism of action, and chemical structure of the most common drugs used in the Ct infection treatment [45,46,47,48,49,50,51,52].

Drug	Chemical Structure	Main Information
Azithromycin	** 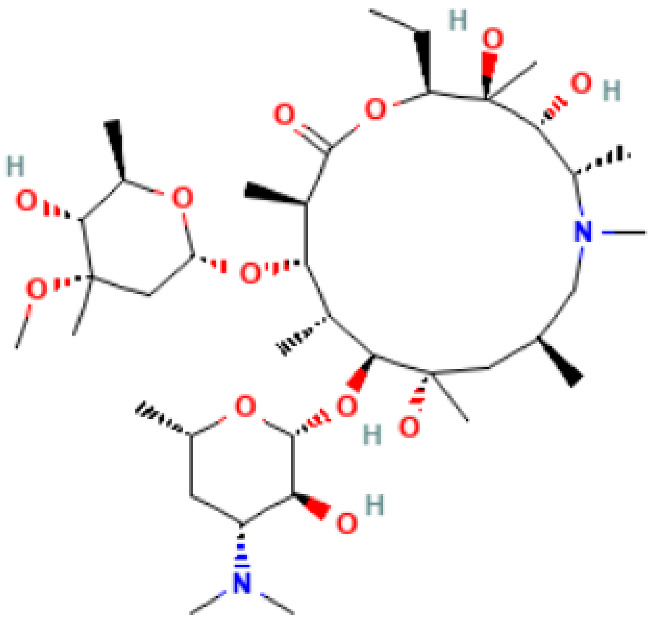 **	Mechanism of action: Inhibition of bacterial protein synthesis by interrupting the transpeptidation/translocation pathway due to its binding to the bacterial 50S ribosomal subunit’s 23S rRNA (ribosomal RNA). Absorption: Following oral administration, peak plasma concentrations occur after 2–3 h. Maximum concentration (C*max*): 0.4 mg.L^−1^). When administered intravenously, peak plasma concentration is reported to be 3–4 mg.L^−1^ after 1 h infusion. Distribution: Mostly distributed in the body (except in the brain and cerebrospinal fluid) the volume of distribution (Vd) is about 31.1 L.kg^−1^. It is well tolerated within cells (phagocytes, e.g.,) allowing high efficacy in Ct infection treatment. Metabolism: Although its metabolic pathway has yet to be explored, it is known that azithromycin undergoes some hepatic metabolism. Route of elimination: Biliary excretion is the major route of elimination; 12.2% of the drug is eliminated in the urine after intravenous (IV) administration and 4.5% when administered orally. Half-life: The elimination half-life (t_1/2_) is 40–68 h due to extensive tissue retention.
Doxycycline	** 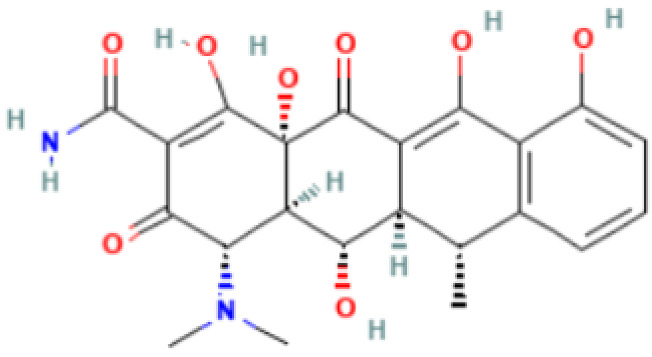 **	Mechanism of action: It prevents aminoacyl-tRNA (aa-tRNA) from binding to the ribosomal site, hence inhibiting bacterial protein synthesis, namely the elongation phase. Absorption: Peak plasma concentration of approximately 3.0–5.0 μg.mL^−1^ occur 2–3 h after oral administration and 4–10 µg.mL^−1^ within 30 min after IV dosing. Distribution: Despite the scarcity of data, it is known that the drug is widely distributed in tissues and body fluids, including cerebrospinal fluid. Metabolism: It has not been studied yet. Route of elimination: Most of the drug is excreted through the kidneys, with a small fraction being eliminated in the bile. It can also be excreted in feces. Half-life: 18–22 h.
Tetracycline	** 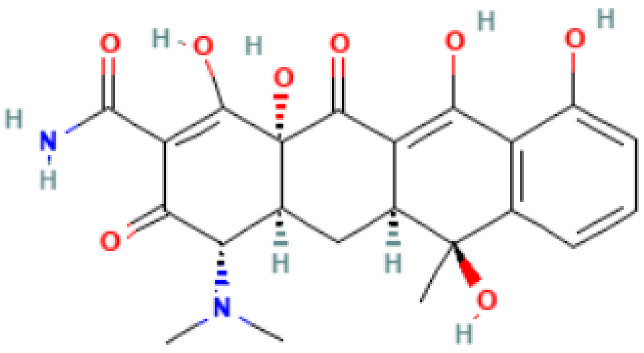 **	Mechanism of action: Inhibition of the ribosome subunits association by binding to the 30S ribosomal subunit via passive diffusion in bacterial membrane porin channels, hence interfering with protein synthesis. Absorption: Following oral administration, peak plasma concentrations of 3–5 μg.mL^−1^ within 2 h. Intramuscular (IM) administration has low bioavailability (<40%), followed by oral (60–80%) and IV administration (100%). Distribution: Limited information available. This drug’s class of antibiotics has a solubility-dependent distribution in the tissues and body fluids. Metabolism: Not metabolized. Route of elimination: It is excreted in the urine (30%) and feces (20–60%) at high concentrations in its biologically active form. Half-life: 6–12 h.
Erythromycin	** 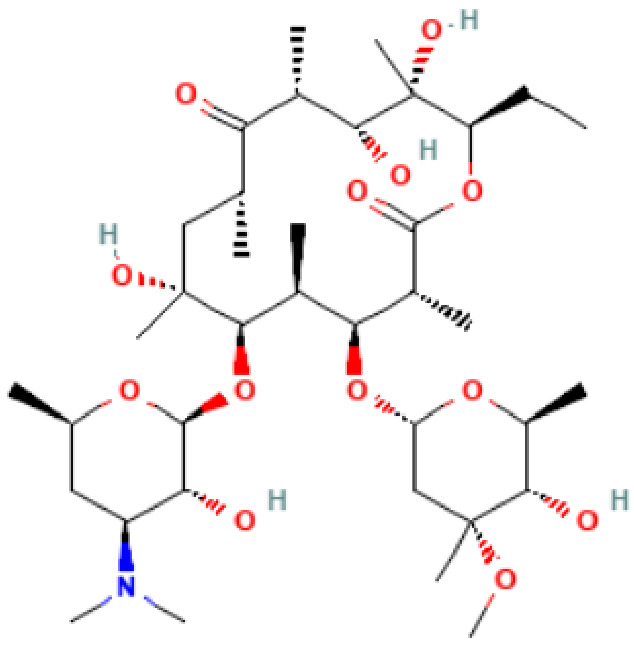 **	Mechanism of action: It inhibits transpeptidation/translocation and the assembly of the 50S ribosomal subunit, preventing bacterial protein synthesis. Absorption: Despite the interindividual heterogeneity in absorption, the peak plasma concentration is reported to be 1.8 μg.L^−1^ after 1.2 h of an orally administered dose. Its bioavailability ranges from 18–45%. Distribution: Found in most body fluids and accumulated in leucocytes and inflammatory liquid (Vd: 1.5 L.kg^−1^). This drug is well diffused in meningitis, as the blood-brain barrier (BBB) is easily penetrated (inflamed tissues). Metabolism: It undergoes hepatic first-pass metabolism after an oral dose. CYP3A4 enzyme partially metabolizes it to N-desmethylerythromycin. In acidic conditions, it is also hydrolyzed to anhydro forms (inactive against bacteria). Route of elimination: It is excreted in the bile. After an oral dosage, less than 5% is eliminated in the urine. Half-life: 2.4–3.1 h.
Levofloxacin	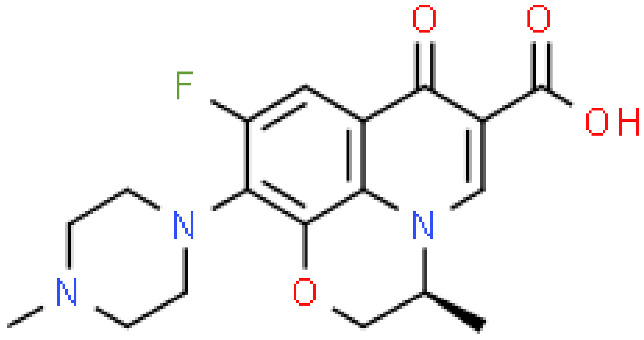	Mechanism of action: It prevents normal cell division by acting on the DNA (deoxyribonucleic acid) gyrase and topoisomerase IV, enzymes responsible for avoiding excessive supercoiling of DNA during replication or transcription. Absorption: Peak plasma concentrations of 11.5 µg.mL^−1^ within 2–3 h following oral administration. Bioavailability is approximately 99%. Distribution: Extensive distribution in body fluids and inflammatory exudates. Vd ranges between 1.09 and 1.26 L.kg^−1^ after an orally administered dose. Metabolism: Levofloxacin metabolism in humans occurs by demethylation and oxidation originating the metabolites: desmethyl-levofloxacin and levofloxacin-*N*-oxide. Route of elimination: After oral administration, approximately 87% is excreted in urine and less than 4% in feces. Half-life: 6–8 h.
Amoxicillin	** 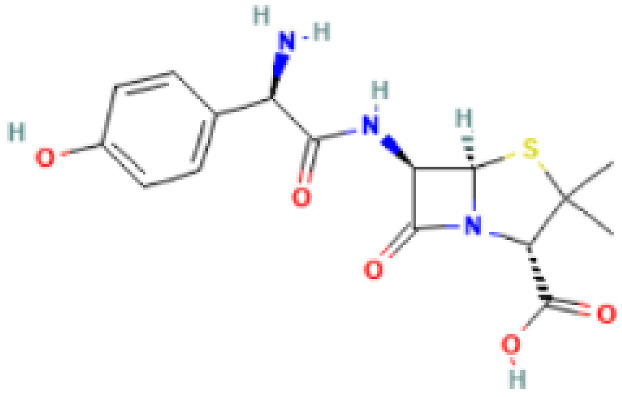 **	Mechanism of action: It inhibits penicillin-binding proteins, which are responsible for glycosyltransferase and transpeptidase reactions that lead to cross-linking of D-alanine and D-aspartic acid in bacterial cell walls. This affects the formation and repair of the cell wall, resulting in cell lysis. Absorption: A 250 mg of oral dose reaches peak plasma concentrations of 3.93 mg.L^−1^ after 1.31 h. Bioavailability is approximately 60%. Distribution: Distribution into liver, lungs, prostate, muscle, and bone is reported in several studies. Vd has been measured to be 27.7 L. Metabolism: It has several metabolic pathways, from hydroxylation, oxidative deamination to decarboxylation. Route of elimination: 70–78% of the drug is eliminated in the urine. Half-life: 1 h.
Chloramphenicol	** 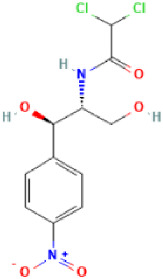 **	Mechanism of action: It binds to the L16 protein of the 50S ribosomal subunit, preventing the transfer of amino acids to growing peptide chains and subsequent protein synthesis. Absorption: Topical application to the eye may also be intraocular and little systemic absorption. Distribution: It has no volume of distribution. Metabolism: It is not metabolized. Route of elimination: Not very clear information. Half-life: 1.5–3.5 h.
Povidone-iodine	** 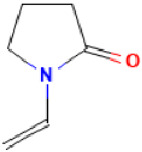 **	Mechanism of action: It is a complex that gradually releases free iodine at the application site. Free iodine penetrates the cell wall, resulting in disruption of protein and nucleic acid structure and synthesis. Absorption: Topical application; it is not absorbed. Distribution: It has no volume of distribution. Metabolism: It is not metabolized. Route of elimination: It is not eliminated. Half-life: Not applicable.
Silver Nitrate	** 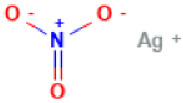 **	Pharmacokinetics information is not available.

**Table 3:** Ct infection treatment options following the guidelines [20,57,58].

Type of Ct Infection	Treatment Options
Uncomplicated genital chlamydia	Doxycycline 100 mg orally twice a day for 7 days Azithromycin 1 g orally as a single dose
	Tetracycline 500 mg orally four times a day for 7 days
	Erythromycin 500 mg orally four times a day for 7 days
	Levofloxacin 500 mg orally once daily for 7 days
Anorectal chlamydial infection	Doxycycline 100 mg orally twice a day for 7 days over
	Azithromycin 1 g orally as a single dose
Genital chlamydial infection in pregnant women	Azithromycin 1 g orally as a single dose
	Amoxicillin 500 mg orally three times a day for 7 days
	Erythromycin 500 mg orally four times a day for 7 days
Lymphogranuloma venereum (LGV)	Doxycycline 100 mg orally twice daily for 21 days Azithromycin 1 g orally, weekly for 3 weeks Erythromycin 500 mg orally four times a day for 21 days
Ophthalmia neonatorum	
*Conjunctivitis*	Azithromycin 20 mg/kg/day orally, one dose daily for 3 days
	Erythromycin 50 mg/kg/day orally, in four divided doses daily for 14 days
*Ocular prophylaxis*	Tetracycline hydrochloride 1% eye ointment
	Erythromycin 0.5% eye ointment
	Povidone iodine 2.5% solution
	Silver nitrate 1% solution
	Chloramphenicol 1% eye ointment

## Data Availability

Not applicable.
